# An exploratory study of different definitions and thresholds for lumbar disc degeneration assessed by MRI and their associations with low back pain using data from a cohort study of a general population

**DOI:** 10.1186/s12891-020-03268-4

**Published:** 2020-04-17

**Authors:** Line Dragsbæk, Per Kjaer, Mark Hancock, Tue Secher Jensen

**Affiliations:** 1 Department of Sports Science and Clinical Biomechanics, University of Southern Denmark, Campusvej 55, 5230 Odense, Denmark; 2 Health Sciences Research Centre, UCL University College, Odense, Denmark; 3 Faculty of Medicine and Health Sciences, Macquarie University, Sydney, Australia; 4 Department of Diagnostic Imaging, Silkeborg Regional Hospital, Silkeborg, Denmark; 5 Department of Clinical Medicine, Aarhus University, Aarhus, Denmark; 6 Nordic Institute of Chiropractic and Clinical Biomechanics, Odense, Denmark

**Keywords:** Lumbar disc degeneration, Low back pain, Magnetic resonance imaging

## Abstract

**Background:**

Lumbar disc degeneration seen on magnetic resonance imaging (MRI) is defined as loss of signal intensity and/or disc height, alone or in combination with other MRI findings. The MRI findings and thresholds used to define disc degeneration vary in the literature, and their associations with low back pain (LBP) remain uncertain.

**Objective:**

To explore how various thresholds of lumbar disc degeneration alter the association between disc degeneration and self-reported LBP.

**Methods:**

An exploratory, cross-sectional cohort study of a general population. Participants in the cohort ‘Backs-on-Funen’ had MRI scans and completed questionnaires about LBP at ages 41, 45 and 49 years. The MRI variables, signal intensity (Grades 0–3) and disc height (Grades 0–3), were dichotomised at different thresholds. Logistic regression analyses were used to determine associations. Arbitrarily, a difference in odds ratio (OR) of > 0.5 between thresholds was considered clinically relevant. Receiver Operating Characteristic curves were used to investigate differences between diagnostic values at each threshold.

**Results:**

At age 41, the difference in ORs between signal loss and LBP exceeded 0.5 between the thresholds of ≥2 (OR = 2.02) and = 3 (OR = 2.57). Difference in area under the curves (AUC) was statistically significant (*p* = 0.02). At ages 45 and 49, the difference in ORs exceeded 0.5 between the thresholds of ≥2 and = 3, but the differences between AUC were not statistically significant. At age 41, the difference in ORs between disc height loss and LBP at the thresholds of ≥1 (OR = 1.44) and ≥ 2 (OR = 2.53) exceeded 0.5. Differences in AUC were statistically significant (*p* = 0.004). At age 49, differences in ORs exceeded 0.5 (OR = 2.49 at the ≥1 threshold, 1.84 at ≥2 and 0.89 at =3). Differences between AUC were not statistically significant.

**Conclusion:**

The results suggest that the thresholds used to define the presence of lumbar disc degeneration influence how strongly it is associated with LBP. Thresholds at more severe grades of disc signal and disc height loss were more strongly associated with LBP at age 41, but thresholds at moderate grades of disc degeneration were most strongly associated with LBP at ages 45 and 49.

## Background

Low back pain (LBP) is a common health problem that results in a large global burden both personally and financially [1]. A global review of the prevalence of LBP in general adult populations found a 1-year prevalence of 38% [2]. Understanding of the causes of LBP remains limited.

Uncertainty exists about the relationship between disc degeneration (DD) identified on MRI and pain. In a meta-analysis by Brinjikji et al., the association between lumbar disc degeneration (LDD) and LBP was investigated in adults aged 15–50 years. An overall odds ratio (OR) of 2.24 (95% confidence interval (CI) 1.21; 4.15) was found with substantial variabilities between the included studies.

Different definitions of LDD in the literature may have affected the reported associations between LDD and LBP, as well as explain the inconsistency in associations across studies. A LDD diagnosis on MRI may be based on one or a combination of abnormal findings (e.g. loss of signal intensity alone or a combination of loss of signal intensity, loss of disc height, inhomogeneous structure of the disc and reduced distinction between nucleus and annulus in the disc). A review by Kettler et al. [3] found five different grading systems [4–8] for defining LDD on MRI, the most common being the one provided by Pfirrmann et al. [8]. In addition to the different definitions of LDD, different thresholds for dichotomising LDD have been used in studies examining the association between LDD and LBP and are likely to affect both the reported prevalence of LDD and its association with LBP.

A systematic search of the literature was conducted to review definitions and thresholds of LDD defined by MRI and their associations with LBP in the literature (Additional file 1).

Twenty studies were included and are presented in a forest plot (Additional File 2), which shows a generally positive association between LDD and LBP. With regard to the LDD definitions, 12 of the 20 studies used Pfirrmann et al.’s definition of LDD [9–20] and eight of the 20 studies used loss of signal intensity as the definition of LDD [21–28]. The thresholds for positive findings are shown in Additional File 2. Three studies investigated associations using different thresholds on the Pfirrmann grading system and are therefore represented more than once in the forest plot [9, 14, 18]. Associations between LDD and LBP varied substantially across the 20 studies. Overall, disc signal intensity was positively associated with LBP: OR 2.13 (95% CI 1.76; 2.58) and disc height was positively associated with LBP: OR 2.58 (CI 1.70; 3.92). The variation in associations was not surprising given the inconsistent definitions and thresholds (Additional files 2 and 3). Study design, number of participants and definition of the LBP outcome may also have contributed to the different associations between LDD and LBP.

Knowledge about how different grades of LDD on MRI are associated with LBP would help clinicians communicate a clearer message to patients about the relevance of MRI findings. It is essential to understand, which degrees of degeneration are related to pain and which are not; and if thresholds are consistent across different ages and for men and women separately.

Using data from a cohort study of a general population, this exploratory study provides a unique possibility to explore different thresholds of LDD in the most valid way, because thresholds are compared within a single sample, including the use of a consistent definition of LBP. Therefore, the aim of this study was to explore how various definitions or thresholds of LDD alter the association between LDD and self-reported LBP.

## Methods

### Study design

The study was an exploratory cross-sectional cohort study of a general population. The data analyses were based on data from the cohort ‘Backs on Funen’, some of which have previously been published. Reporting of this study follows the strengthening of reporting of observational studies in epidemiology (STROBE) statement [29].

### Study participants and sampling from ‘Backs on Funen’

Participants were selected from the population of the county of Funen, Denmark, in the year 2000. They were selected from the Central Office of Civil Registration, where every ninth person was selected of those born in Denmark during the period of May 27, 1959, to May 26, 1960, and living in the county of Funen in June 2000 [25]. We assumed that when sampling every ninth person of this age-specific cohort, we would have a random sample with relative representation of all districts, as well as potential stratums and blocks. Furthermore, we did analyse the representativeness of our sample against the county of Funen and the Danish population and found our sample to be fairly comparable with a slight under-representation of people with only basic education and a slight overrepresentation of people with long-cycle higher education and top managers [30, 31].

### Study procedures of ‘Backs on Funen’

The selected individuals received information about the study by mail and agreed to participate by responding to the mail. Participants were examined first time from June 2000 to February 2001 at the Back Research Center in Ringe, Funen, Denmark. Participants were 41 years at the time of their first visit, during which a lumbar MRI scan and a clinical examination of the lumbar spine were performed. The clinical examination included analyses of posture, active movements of the lumbar spine, motor control, palpation, pain provocation tests and lifting capacity [32]. Participants also completed a set of questionnaires about their LBP. At the second time point, participants were 45 years old, and at the third time point, 49 years old. At both follow-up time points, participants had an MRI and again completed the questionnaires. Details about the study procedures have been published elsewhere [25, 33]. A radiologist, who was blinded to any other information about the participants, reported on the MRIs, using a standardised protocol [25].

### MRI procedures for ‘Backs on Funen**’**

MRI was performed with an open, low-field 0.2 T magnetic resonance unit (Magnetom Open Viva, Siemens AG, Erlangen, Germany). A detailed description of the MRI procedures has been reported previously [25].

### LBP variable

The LBP questionnaires contained questions that had been partially validated and used in other Scandinavian studies [34–36]. For the purpose of the current study, the outcome variable ‘LBP year’ was used, and defined as “Yes” to the question: “Have you had trouble with the lowest part of your back (picture provided) during the past 12 months?” [33].

### Definitions of MRI variables

In the existing literature, disc signal intensity and disc height were the two most commonly used MRI findings to define LDD, either alone or in combination. Therefore, signal intensity and disc height were chosen as the two MRI variables used to define LDD in the current study. Dehydration of the disc results in a loss of signal intensity on MRI, which is reflected in darkening of the disc on the image. Signal intensity was scored on a 0–3 point scale, where Grade 3 represented the most severe loss of signal intensity of the disc (Grade 0: Homogeneously hyperintense, Grade 1: Hyperintense with visible intranuclear cleft, Grade 2: Intermediate signal intensity, Grade 3: Hypointense) [37]. For analysis, signal intensity was dichotomised at the thresholds of ≥1, ≥ 2 and = 3 on the 0–3-point scale.

Degenerated *discs* lose *height*, and the distance between vertebrae is reduced. Disc height was scored on a 0–3 point scale, describing the height of the discs and the distance between vertebrae. Grade 3 represented the most severe degree of disc height loss (Grade 0: Disc higher than the disc above, Grade 1: Disc as high as the disc above (if normal), Grade 2: Disc narrower than the disc above (if normal), Grade 3: Endplates almost in contact) [38]. For analysis, disc height was dichotomised at the thresholds of ≥1, ≥ 2 and = 3 on the 0–3-point scale. Kappa values for intra- and inter-observer reliability for disc signal and disc height were 0.87 (0.77–0.97) / 0.59 (0.50–0.68) and 0.81 (0.71–0.91) / 0.66 (0.56–0.76), respectively [39]. For each participant, the score from the spinal level with the worst degree of signal intensity loss and the level with the worst degree of disc height loss were reported and used in the regression analyses.

### Other variables

As being overweight and obese are known to be risk factors for LBP [40], body mass index (BMI) was included as a potential confounder. BMI was calculated on the basis of the participant’s self-reported height and weight (weight divided by height squared). The prevalence of LBP and LDD differs between sexes and instead of adjusting for sex, we explored the results in males and females separately.

### Statistical analyses

Normal distribution of BMI was tested using normal probability plots. Comparison of mean BMI in the groups with LBP and no-LBP was performed using the student’s t-test. Differences in distribution of sex and MRI findings between the groups with LBP and no-LBP were tested using Pearson’s chi-squared test where expected cell counts were > 5. If expected cell counts were < 5, Fischer’s exact test was used to test difference in distribution. Cross-sectional associations between LDD and LBP at different thresholds were investigated at the three time points (41 years, 45 years and 49 years). Logistic regression analyses for each threshold of DD were performed to explore associations with LBP. As a possible confounder, BMI was included in the logistic regression analyses as a continuous variable. Because of the exploratory nature of the study and the focus on the impact of changed thresholds on association with pain, no other confounders were included. Associations between LDD and LBP at each threshold were expressed as OR with 95% CI. The underlying assumption of linearity between independent variables and log odds was tested using lowess plots. Pre-hoc, we set an arbitrary difference in OR of > 0.5 between thresholds as clinically relevant.

To further explore whether the differences in diagnostic accuracy of the thresholds were statistically significant, Receiver Operating Characteristic (ROC) curves were constructed and compared using the STATA command roccomp, which produces a chi-square test. Secondary analyses were performed separately for each sex and by location of LDD in the lumbar spine (upper lumbar spine = L1-L2 and lower lumbar spine = L3-L5). All statistical analyses were performed using STATA/IC 15.0 (StataCorp, College Station, Tx, USA).

### Missing data

Cases with missing values on any variable used in the analyses were dropped.

## Results

### Study participants

In total, 412 of the 625 people invited, agreed to participate at the first time point (41 years old). One hundred and ninety-nine (48.30%) were men, and 213 (51.70%) were women. Three hundred and forty-eight people participated (83% of the baseline population) 4 years later (45 years old), 161 (46.26%) were men, and 187 (53.74%) were women. Eight years after baseline (49 years old), 293 people participated (70.9% of the baseline population). Of these, 136 (46.42%) were men and 157 (53.58%) were women (Table 1).

**Table 1:** Participant characteristics and prevalence of MRI findings in LBP and no-LBP groups including test for group difference. The MRI findings are based on the worst score at any lumbar level (L1-L5)

41 Years				
Characteristics	Total ***n*** = 412	LBP Year ***n*** = 284 (68.93%)	No LBP Year ***n*** = 128 (31.07%)	***P***-Value
Sex				
Men n (%)	199 (48.30)	134 (47.18)	65 (50.78)	0.499
Women n (%)	213 (51.70)	150 (52.82)	63 (49.22)	
BMI Mean (SD)	25.05 (4.25)	25.24 (4.28)	24.63 (4.19)	0.176
MRI Findings				
Signal Intensity (worst lumbar level)				
Grade 0 n (%)	4 (0.97)	3 (1.06)	1 (0.78)	
Grade 1 n (%)	51 (12.38)	28 (9.86)	23 (17.97)	
Grade 2 n (%)	171 (41.50)	105 (36.97)	66 (51.56)	
Grade 3 n (%)	186 (45.15)	148 (52.11)	38 (29.69)	0.000*^a^
Disc Height (worst lumbar level)				
Grade 0 n (%)	90 (21.84)	56 (19.72)	34 (26.56)	
Grade 1 n (%)	103 (25.00)	57 (20.07)	46 (35.94)	
Grade 2 n (%)	207 (50.24)	161 (56.69)	46 (35.94)	
Grade 3 n (%)	12 (2.91)	10 (3.52)	2 (1.56)	0.000*^a^
**45 Years**				
**Characteristics**	**Total** ***n*** **= 348**	**LBP Year** ***n*** **= 238 (68.39%)**	**No LBP Year** ***n*** **= 110 (31.61%)**	***P*-Value**
Sex				
Men n (%)	161 (46.26)	109 (45.80)	52 (47.27)	0.798
Women n (%)	187 (53.74)	129 (54.20)	58 (52.73)	
BMI Mean (SD)	25.16 (4.05)	25.43 (4.23)	24.57 (3.60)	0.051
MRI Findings				
Signal Intensity (worst lumbar level)				
Grade 0 n (%)	0	0	0	
Grade 1 n (%)	52 (15.12)	28 (11.81)	24 (22.43)	
Grade 2 n (%)	121 (35.17)	89 (37.55)	32 (29.91)	
Grade 3 n (%)	171 (49.71)	120 (50.63)	51 (47.66)	0.033*^b^
Disc Height (worst lumbar level)				
Grade 0 n (%)	107 (31.10)	73 (30.80)	34 (31.78)	
Grade 1 n (%)	69 (20.06)	48 (20.25)	21 (19.63)	
Grade 2 n (%)	154 (44.77)	109 (45.99)	45 (42.06)	
Grade 3 n (%)	14 (4.07)	7 (2.95)	7 (6.54)	0.458^a^
**49 Years**				
**Characteristics**	**Total** ***n*** **= 293**	**LBP Year** ***n*** **= 204 (69.62%)**	**No LBP Year** ***n*** **= 89 (30.38%)**	***P*-Value**
Sex				
Men n (%)	136 (46.42)	85 (41.67)	51 (57.30)	0.014*
Women n (%)	157 (53.58)	119 (58.33)	38 (42.70)	
BMI Mean (SD)	27.28 (17.84)	28.19 (21.21)	25.21 (3.44)	0.054
MRI Findings				
Signal Intensity (worst lumbar level)				
Grade 0 n (%)	0	0	0	
Grade 1 n (%)	31 (10.88)	15 (7.54)	16 (18.60)	
Grade 2 n (%)	154 (54.04)	109 (54.77)	45 (52.33)	
Grade 3 n (%)	100 (35.09)	75 (37.69)	25 (29.07)	0.017*^b^
Disc Height (worst lumbar level)				
Grade 0 n (%)	44 (15.44)	23 (11.56)	21 (24.42)	
Grade 1 n (%)	52 (18.25)	36 (18.09)	16 (18.60)	
Grade 2 n (%)	160 (56.14)	120 (60.30)	40 (46.51)	
Grade 3 n (%)	29 (10.18)	20 (10.05)	9 (10.47)	0.037*^b^

* = Statistically significant difference between LBP and no-LBP groups (*P*-value < 0.05)

^a^ = *P*-value was obtained using Fischer’s exact test

^b^ = P-value was obtained using Pearson’s chi2 test

A majority of individuals reported pain during the last 12 months at each of the follow up time points. Of all the individuals, 68.93% (41 years), 68.39% (45 years), and 69.62% (49 years) answered “yes” to having had LBP during the last 12 months (Table 1). BMI data was estimated to be normally distributed.

### Missing data

Three individuals had missing data on BMI and were not included in the regression analyses. Therefore, the data of 409 individuals were used in the overall regression analyses at 41 years, 345 at 45 years, and 290 at 49 years.

### Prevalence of MRI findings

The prevalence of MRI findings in the LBP and no-LBP groups are presented in Table 1.

For signal intensity, few individuals at 41 years, and none at 45 years were assessed as Grade 0. Across the included population (regardless of pain history), the most prevalent grades for signal intensity at the worst spinal level were Grades 2 and 3.

For disc height, Grade 2 was the most prevalent grade at the worst spinal level across the whole population. Grade 3 (endplates almost in contact) was not very prevalent, as it only constituted between 2 and 10% of the grades in each subgroup (LBP and no-LBP).

### Association between disc signal intensity and LBP

Normal distribution of data and the underlying assumption of linearity in the logistic regression analyses was confirmed. As shown in Table 2, statistically significant positive associations were found between loss of signal intensity and LBP at the threshold of ≥2 at all three ages. For the threshold of =3, the association with LBP was only significant at the age of 41. The results for the threshold of ≥1 (signal intensity) were not presented in Table 2, as very few individuals had a signal intensity score of grade 0, meaning that there were approximately 1% of cases in the comparison (reference) group. The strength of association (ORs) between thresholds of > 2 and = 3 differed by more than our prespecified level of 0.5 at all age groups (2.02 vs 2.57 at age 41, 2.17 vs 1.10 at age 45 and 2.65 vs 1.49 at age 49). However, the difference between Area Under the Curve (AUC) of the receiver operating curve for the two thresholds (> 2 vs =3) was only statistically significant at age 41 (*p* = 0.020) and not at ages 45 or 49. In addition, the association was strongest for the threshold of =3 at age 41, but at the ages of 45 and 49, the association was strongest using the threshold of > 2. AUC values for signal intensity are presented in Additional file 4.

**Table 2:** Associations and difference in diagnostic accuracy between disc signal intensity and ‘LBP year’ at different thresholds and ages. Associations are expressed as odds ratios (OR) with 95% confidence intervals (95% CI) obtained from logistic regression analyses adjusted for body mass index. The probability of no difference in Area Under Curves (AUC) between Thresholds 1 and 2 is reported as *p*-values from chi square tests. More details are given in Additional File 4

Disc signal intensity at 41 Years	Threshold 2: ≥ grade 2 (*n* = 356) (87.04%) OR (95% CI)	Threshold 3: = grade 3 (*n* = 185) (45.23%) OR (95% CI)	AUC comparison (*p*-value)
**All participants (*n*** **= 409)**	2.02 (1.12;3.64)*	2.57 (1.65;4.02)*	AUC T2 = AUC T3: 0.020*
Men (*n* = 197)	2.88 (1.20;6.90)*	2.52 (1.35;4.72)*	AUC T2 = AUC T3: 0.017*
Women (*n* = 210)	1.63 (0.70;3.76)	2.90 (1.50;5.60)*	AUC T2 = AUC T3: 0.016*
Upper lumbar spine (n = 409)	1.16 (0.76;1.77)	2.27 (0.91;5.62)	AUC T2 = AUC T3: 0.240
Lower lumbar spine (n = 409)	1.91 (1.16;3.16)*	2.55 (1.61;4.05)*	AUC T2 = AUC T3: 0.111
**Disc signal intensity at 45 Years**	Threshold 2: ≥ grade 2 (*n* = 290) (85.04%) OR (95% CI)	Threshold 3: = grade 3 (*n* = 170) (49.85%) OR (95% CI)	AUC comparison (*p*-value)
**All participants (*n*** **= 341)**	2.17 (1.18;4.00)*	1.10 (0.69;1.75)	AUC T2 = AUC T3: 0.123
Men (*n* = 160)	2.25 (0.92;5.47)	1.47 (0.75;2.90)	AUC T2 = AUC T3: 0.134
Women (*n* = 181)	2.11 (0.90;4.92)	0.85 (0.45;1.61)	AUC T2 = AUC T3: 0.267
Upper lumbar spine (n = 341)	1.34 (0.84;2.14)	1.48 (0.72;3.06)	AUC T2 = AUC T3: 0.767
Lower lumbar spine (n = 341)	1.57 (0.91;2.74)	0.98 (0.61;1.55)	AUC T2 = AUC T3: 0.589
**Disc signal intensity at 49 Years**	Threshold 2: ≥ grade 2 (*n* = 251) (89.01%) OR (95% CI)	Threshold 3: = grade 3 (*n* = 100) (35.46%) OR (95% CI)	AUC comparison (*p*-value)
**All participants (*n*** **= 282)**	2.65 (1.23;5.67)*	1.49 (0.86;2.59)	AUC T2 = AUC T3: 0.859
Men (*n* = 132)	5.49 (1.63;18.52)*	2.30 (1.05;5.06)*	AUC T2 = AUC T3: 0.825
Women (*n* = 150)	1.35 (0.43;4.25)	0.98 (0.44;2.16)	AUC T2 = AUC T3: 0.01*
Upper lumbar spine (n = 282)	2.08 (1.21;3.58)*	5.08 (1.16;22.21)*	AUC T2 = AUC T3: 0.247
Lower lumbar spine (n = 282)	2.23 (1.15;4.30)*	1.29 (0.74;2.26)	AUC T2 = AUC T3: 0.324

Note: The results for threshold ≥1 were underpowered and are therefore not presented in the Table

* *P*-value < 0.05

Secondary analyses stratified by sex and location of LDD in the lumbar spine did not reveal any noticeable and clear trends in differences in associations at the different thresholds for signal intensity (Table 2).

### Association between disc height and LBP

As shown in Table 3, statistically significant associations between loss of disc height and LBP were found for the threshold of ≥2 at age 41 and 49 and for the threshold of ≥1 at age 49.

**Table 3:** Associations and difference in diagnostic accuracy between disc height and ‘LBP year’ at different thresholds and ages. Associations are expressed as odds ratios (OR) with 95% confidence intervals (95% CI) obtained from logistic regression analyses adjusted for body mass index. The probability of no difference in Area Under Curves (AUC) between Thresholds 1, 2 and 3 is reported as *p*-values from chi square tests. More details are given in Additional File 5

Disc height at 41 Years	Threshold 1: ≥ grade 1 (*n* = 319) (77.99%) OR (95% CI)	Threshold 2: ≥ grade 2 (*n* = 218) (53.30%) OR (95% CI)	Threshold 3: = grade 3 (*n* = 12) (2.93%) OR (95% CI)	AUC comparison (*p*-value)
**All participants (*n*** **= 409)**	1.44 (0.89;2.35)	2.53 (1.64;3.90)*	2.29 (0.49;10.63)	AUC T1 = AUC T2: 0.004* AUC T2 = AUC T3: 0.006* AUC T1 = AUC T3: 0.760
Men (*n* = 197)	2.14 (1.02;4.49)*	3.96 (2.11;7.43)*	1.82 (0.20;16.80)	AUC T1 = AUC T2: 0.001* AUC T2 = AUC T3: 0.000* AUC T1 = AUC T3: 0.121
Women (*n* = 210)	1.09 (0.55;2.14)	1.73 (0.94;3.18)	2.12 (0.24;18.55)	AUC T1 = AUC T2: 0.001* AUC T2 = AUC T3: 0.004* AUC T1 = AUC T3: 0.586
Upper lumbar spine (*n* = 409)	1.44 (0.88;2.36)	3.68 (1.62;8.38)*	–	AUC T1 = AUC T2: 0.097
Lower lumbar spine (*n* = 409)	1.49 (0.94;2.38)	2.31 (1.49;3.57)*	2.00 (0.42;9.42)	AUC T1 = AUC T2: 0.019 AUC T2 = AUC T3: 0.01* AUC T1 = AUC T3: 0.519
**Disc height at 45 Years**	Threshold 1: ≥ grade 1 (*n* = 237) (69.50%) OR (95% CI)	Threshold 2: ≥ grade 2 (*n* = 168) (49.27%) OR (95% CI)	Threshold 3: = grade 3 (*n* = 14) (4.11%) OR (95% CI)	AUC comparison (*p*-value)
**All participants (*n*** **= 341)**	1.05 (0.64;1,74)	0.97 (0.61;1,55)	0.43 (0.15;1.26)	AUC T1 = AUC T2: 0.724 AUC T2 = AUC T3: 0.296 AUC T1 = AUC T3: 0.275
Men (*n* = 160)	1.53 (0.73;3.21)	1.17 (0.59;2.29)	1.13 (0.21;6.09)	AUC T1 = AUC T2: 0.725 AUC T2 = AUC T3: 0.954 AUC T1 = AUC T3: 0.795
Women (*n* = 181)	0.77 (0.39;1.55)	0.83 (0.43;1.58)	0.16 (0.03;0.87)*	AUC T1 = AUC T2: 0.929 AUC T2 = AUC T3: 0.441 AUC T1 = AUC T3: 0.540
Upper lumbar spine (*n* = 341)	1.51 (0.86;2.64)	1.94 (0.93;4.06)	–	AUC T1 = AUC T2: 0.735
Lower lumbar spine (*n* = 341)	0.99 (0.62;1.60)	0.96 (0.60;1.54)	0.43 (0.15;1.26)	AUC T1 = AUC T2: 0.896 AUC T2 = AUC T3: 0.284 AUC T1 = AUC T3: 0.302
**Disc height at 49 Years**	Threshold 1: ≥ grade 1 (*n* = 238) (84.40%) OR (95% CI)	Threshold 2: ≥ grade 2 (*n* = 187) (66.31%) OR (95% CI)	Threshold 3: = grade 3 (*n* = 29) (10.28%) OR (95% CI)	AUC comparison (*p*-value)
**All participants (*n*** **= 282)**	2.49 (1.28;4.85)*	1.84 (1.08;3.13)*	0.89 (0.38;2.06)	AUC T1 = AUC T2: 0.995 AUC T2 = AUC T3: 0.134 AUC T1 = AUC T3: 0.070
Men (*n* = 132)	3.22 (1.27;8.16)*	2.20 (1.04;4.64)*	1.73 (0.51;5.84)	AUC T1 = AUC T2: 0.864 AUC T2 = AUC T3: 0.046* AUC T1 = AUC T3: 0.019*
Women (*n* = 150)	1.69 (0.61;4.68)	1.57 (0.72;3.42)	0.44 (0.13;1.48)	AUC T1 = AUC T2: 0.876 AUC T2 = AUC T3: 0.081 AUC T1 = AUC T3: 0.074
Upper lumbar spine (*n* = 282)	3.69 (2.00;6.81)*	4.77 (2.17;10.49)*	–	AUC T1 = AUC T2: 0.448
Lower lumbar spine (*n* = 282)	1.74 (0.95;3.17)	1.50 (0.89;2.52)	0.89 (0.38;2.10)	AUC T1 = AUC T2: 0.971 AUC T2 = AUC T3: 0.233 AUC T1 = AUC T3: 0.202

* = *P*-value < 0.05

-No participants with or without LDD at this threshold

The strength of association (ORs) between the thresholds of > 1 and ≥ 2 differed by more than our prespecified level of 0.5 at age 41 (1.44 vs 2.53). The difference between AUC for the two thresholds (> 1 vs ≥2) was statistically significant at this age (*p* = 0.004).

The strength of positive associations between the thresholds of ≥1 and ≥ 2 and between ≥2 and = 3 differed more than 0.5 at age 49 (2.49 at ≥1, 1.84 at ≥2 and 0.89 at =3). The differences in AUCs between thresholds were not statistically significant at ages 45 and 49. In spite of a difference under 0.5 in ORs between the thresholds of ≥2 and = 3 (2.53 vs 2.29) at 41 years, there was a statistically significant difference in AUCs at this age (*p* = 0.006). AUC values for disc height are presented in Additional file 5.

Stratifying by sex and location of LDD did not reveal new trends as to which threshold had the strongest association with LBP (Table 3). However, at almost every threshold, stronger associations were seen for men than for women.

## Discussion

### Summary of main findings

This exploratory study investigated the associations between various definitions of LDD defined by MRI at different thresholds and self-reported LBP in a cohort from the general Danish population. At age 41, signal intensity of =3 was more strongly associated with LBP than signal intensity of ≥2, but at age 45 and 49 it was the opposite. The differences in ORs exceeded 0.5. A disc height of ≥2 was more strongly associated with LBP than a disc height of ≥1 at age 41. At age 49, a disc height of ≥1 was more strongly associated with LBP than a disc height of ≥2 and further, a disc height of ≥2 was more strongly associated with LBP than a disc height of =3. The mentioned differences in ORs exceeded 0.5.

### Interpretation

The current study showed a stronger association between LDD and pain, at the thresholds of more severe grades of signal intensity loss than at moderate grades at age 41. At age 49 thresholds at more moderate grades of signal intensity loss seemed to be more strongly associated with LBP. Other studies, which have used loss of disc signal intensity to define LDD, have reported varying associations with LBP (Additional file 2), and those that used a higher threshold did not show any consistent tendency towards stronger associations than those that used a lower threshold [21, 25, 26, 28].

Two of the studies [10, 25] that met our inclusion criteria for the review (Additional files 2 and 3), examined associations between disc height loss and LBP. Hancock et al. 2015 [10] found a Hazard Ratio for recurrence of LBP of 3.24 (1.0;10.52) in a prospective cohort study. In this study, a threshold of ≥1 on a 0–3 scale was used. In our study, statistically significant ORs were only found for the threshold of ≥1 and ≥ 2, and statistically significant and clinically meaningful associations seemed to be stronger at the threshold of ≥2 than =3. This could raise the hypothesis that discs are more painful in the phase of moderate degeneration, than they are when they are more severely degenerated and more fibrotic. This hypothesis was examined by Bendix et al. [21], using disc signal intensity as the definition of LDD. However, the hypothesis was not confirmed. Our study provides preliminary evidence that severely degenerated discs may be less painful than those with only moderate signs of degeneration, at least at ages older than 41. Our results suggest that associations between LDD and LBP may not be consistent at all ages. Further studies that investigate associations between different degrees of LDD and LBP at different ages must be performed to support this hypothesis. Our secondary analysis showed a tendency towards clinically stronger associations between loss of disc height and LBP for men than for women. This could be a spurious finding due to lack of power or methodological limitations. It could also be explained by the fact that different MRI findings affect men and women differently or that other findings or psychological or social factors play a greater role in women than in men in the explanation of LBP. Another explanation could be that men in general have more physically demanding jobs including e.g. heavy lifting. The relationship between heavy lifting and prevalence of LDD is, however, uncertain [41–43].

Most studies investigating LDD use the 5-point scale Pfirrmann grading system [8], which uses a combination of MRI findings to define LDD. Hancock et al. [44] investigated associations at both a threshold of ≥3 and ≥ 4 Pfirrmann grades, and concluded that the threshold used to classify LDD strongly influences the relationship between LDD and LBP. It would be important to perform further studies that examine the associations between LDD as defined by Pfirrmann grades at different thresholds and LBP. This was not possible in the current study, as the MRI findings of disc signal and disc height were reported separately and not as a primary rating on the Pfirrmann grading system.

### Limitations

This investigation using data from the cohort ‘Backs on Funen’ provided a unique opportunity to compare associations between different definitions of LDD and LBP, within a single population and using a single outcome. Several of the ORs obtained by logistic regression analyses are associated with uncertainty, evidenced by the broad 95% CIs. Even though statistically significantly differences were seen between AUCs at different thresholds, the AUCs themselves were low (0.46–0.67), suggesting that signal intensity and disc height seen on MRI may be inadequate diagnostic tests to distinguish between individuals with and without LBP. Because of the number of analyses performed, especially secondary analyses, the results must be interpreted with caution because of the risk of type I errors. Furthermore, stratifying by sex and distinguishing between the lumbar levels in the secondary analyses creates a problem with few individuals meeting certain criteria, resulting in imprecision. Some large ORs and confidence intervals may be explained by inadequate case numbers in some analyses and indicate sparse-data bias [45]. One way to avoid the risk of type I error could have been to use the Bonferroni correction. However, as the purpose of this exploratory study was to identify tendencies in the data to guide further studies of this topic, it was decided to keep the alpha level at 0.05.

No pre-hoc sample size calculation was performed. However, based on the general rule of thumb by Green (*N* > 50 + 8 m) [46, 47], the minimum sample size for the regression analyses for the present study was 66, and sample sizes at all three ages was sufficient.

For disc height at age 41, a statistically significant difference in AUCs was found (p= 0.006) between the thresholds of ≥ 2 and = 3. This is surprising given the close and imprecise ORs 2.53 (1.64;3.90) at the threshold of ≥2 vs. 2.29 (0.49;10.63) at the threshold of =3). This can be explained by lack of power at the threshold of =3 in combination with a common outcome. This results in a poor fit of data for our model. No statistical method is available to directly test differences in ORs. Therefore, differences in AUCs were used to support findings of differences in ORs. However, this surrogate method could lead to the contradictory results as mentioned above.

Three individuals had missing data on BMI and were not included analyses. This handling of missing data may have resulted in selection bias, but as the numbers were small, it is unlikely to change the results in any substantial way.

The LBP outcome used in this study may be seen as a weakness as ‘LBP year’ is a very broad definition including all types of LBP, both acute and chronic, mild and severe. However, there is no clear definition of LBP, and the focus of the current study was not on the strength of associations between imaging and LBP, but rather on how the association varied based on the threshold for LDD.

The diagnostic capability of low-field MRI (0.25 T) has been found comparable to that of high-field MRI (1.5 T and 3.0 T) with respect to degenerative findings of the lumbar spine, and it is not expected that the field strength had an influence on the results of the present study [48].

### Implication

The results from this study are based on findings from a population sample, representative of the general Danish population. Since disc degeneration and LBP are very common in general populations, it is likely that our results can be generalised to other populations including patients. It would be interesting to test our findings in older and younger populations, not included in our sample. It is likely that the thresholds most strongly associated with LBP may be different in younger people. For example, even low levels of signal intensity or disc height loss may represent relevant pathology in younger people while they may be normal age-related findings in older people.

Research indicates that diagnostic imaging for LBP is associated with higher medical costs, increased healthcare utilization and more absence from work [49]. This tendency may partly be explained by the lack of knowledge about the normal age-related imaging findings of the spine. Uncertainty about the meaning of disc degeneration seen on MRI may in some cases lead to unnecessary worries for the patient and may support the notion of degenerative changes as a pathological diagnosis. This exploratory study provides knowledge of the aetiology of LBP and hypotheses about the course of disc degeneration. It forms the basis for further investigations, which should aim to provide a more age and sex specific interpretation of disc degeneration seen on MRI. Even though our ultimate intention with this study was to help the practitioner distinguish between painful and non-painful disc degeneration to enable clear communication of clinically important findings to the patient, the results of this study remain a brick in the puzzle and have more immediate research implications than clinical implications.

## Conclusion

The results of this exploratory study suggest that the thresholds used to define the presence of lumbar disc degeneration, and the participant’s age, influenced how strongly lumbar disc degeneration was associated with low back pain. Even though we saw a tendency for disc degeneration at thresholds at more severe grades of disc signal and disc height loss to be more strongly associated with low back pain at age 41, disc degeneration was most strongly associated with low back pain at thresholds at more moderate grades at ages 45 and 49.

## Supplementary information

**Additional file 1.** Review details. Search matrix, search string, inclusion criteria.

**Additional file 2.** Forest plot. Associations between LBP and LDD (Disc Signal Intensity, Pfirrmann’s grade and Disc Height) in 20 studies. Associations are expressed as Odds Ratios with 95% confidence intervals (95% CI).

**Additional file 3.** Included studies. Study characteristics, definitions of LDD, thresholds for LDD and associations with LBP. Table of included studies in the review including study characteristics, definitions of LDD, thresholds for LDD and associations with LBP. (PDF 208 kb)

**Additional file 4.** Accuracy of reduction in disc signal intensity at different thresholds as a predictor of LBP year at ages 41,45 and 49 years. Area under the curve values with 95% confidence intervals as measures of accuracy of reduction in disc signal intensity at different thresholds as a predictor of LBP year at ages 41,45 and 49 years. (PDF 366 kb)

**Additional file 5.** Accuracy of reduction in disc height at different thresholds as a predictor of LBP year at ages 41,45 and 49 years. Area under the curve values with 95% confidence intervals as measures of accuracy of reduction in disc height at different thresholds as a predictor of LBP year at ages 41, 45 and 49 years.

**Additional file 6.** STROBE statement- checklist of items that should be included in reports of observational studies. Completed STROBE checklist.

## Data Availability

The datasets generated and/or analysed during the current study are not publicly available due to the Backs on Funen study regulations, but are available from the corresponding author on reasonable request.

## Abbreviations

MRI: Magnetic Resonance Imaging
LBP: Low Back Pain
DD: Disc Degeneration
LDD: Lumbar Disc Degeneration
OR: Odds Ratio
CI: Confidence Interval
AUC: Area Under the Curve
ROC: Receiver Operating Characteristic
