# Supplementary material for: An exploratory study of different definitions and thresholds for lumbar disc degeneration assessed by MRI and their associations with low back pain using data from a cohort study of a general population
Source: BMC Musculoskelet Disord. 2020 Apr 17;21:253. doi: 10.1186/s12891-020-03268-4 (PMC7165403; doi:10.1186/s12891-020-03268-4)
Supplement: Supplementary file 1 — Additional file 1. Review details. Search matrix, search string, inclusion criteria. [file 12891_2020_3268_MOESM1_ESM.pdf]

Additional file I. Review details

Search matrix:

Focus I:<br>Disc degeneration	Focus II<br>MRI	Focus III<br>Low Back Pain
MeSH	MeSH	MeSH
Intervertebral Disc Degeneration	Magnetic Resonance Imaging	Low Back Pain
Title/Abstract	Title/Abstract	Title/Abstract
”disc degeneration”<br>”disk degeneration”<br>”degenera* disc”<br>”degenera* disk”<br>”intervertebral disc degeneration”<br>”disc changes”<br>”disk changes”	”Magnetic resonance imaging”<br>”Magnetic resonance”<br>”MR imaging”<br>MRI<br>”Diagnostic imaging”<br>”Radiolog* abnormal*”	”Low Back Pain”<br>LBP<br>”Low-back pain”<br>”Back pain”<br>Back-pain<br>Backpain<br>Lumbago

Search String:

PubMed 20.09.2018:

```
((((((((((((((((((((((("disc degeneration"[Title/Abstract]) OR
”disk degeneration"[Title/Abstract]) OR ”degenera*
disc”[Title/Abstract]) OR ”degenera* disk”[Title/Abstract]) OR
"intervertebral disc degeneration"[Title/Abstract]) OR ”disc
changes” [Title/Abstract]) OR ”disk changes” [Title/Abstract]) OR
"Intervertebral Disc Degeneration"[Mesh])) AND ((((((("magnetic
resonance imaging"[Title/Abstract]) OR "magnetic
resonance"[Title/Abstract]) OR "MR imaging"[Title/Abstract]) OR
MRI[Title/Abstract]) OR "diagnostic imaging"[Title/Abstract]) OR
”radiolog* abnormal*”[Title/Abstract]) OR "Magnetic Resonance
Imaging"[Mesh])) AND (((((((("Low Back Pain"[Title/Abstract]) OR
LBP[Title/Abstract]) OR "low-back pain"[Title/Abstract]) OR "back
pain"[Title/Abstract]) OR back-pain[Title/Abstract]) OR
backpain[Title/Abstract]) OR lumbago[Title/Abstract]) OR "Low Back
Pain"[Mesh]))))))))
```

Studies included:

- Prospective-/retrospective cohort studies, case-control studies and cross-sectional studies

Inclusion criteria:

- Cases / non-cases of LBP
- Adults i.e. > 18 years
- Clear definition of LDD on MRI
- Stated threshold for LDD on MRI
- Full text available in English or Danish
