# Supplementary figures and images for: An exploratory study of different definitions and thresholds for lumbar disc degeneration assessed by MRI and their associations with low back pain using data from a cohort study of a general population
Source: BMC Musculoskelet Disord. 2020 Apr 17;21:253. doi: 10.1186/s12891-020-03268-4 (PMC7165403; doi:10.1186/s12891-020-03268-4)

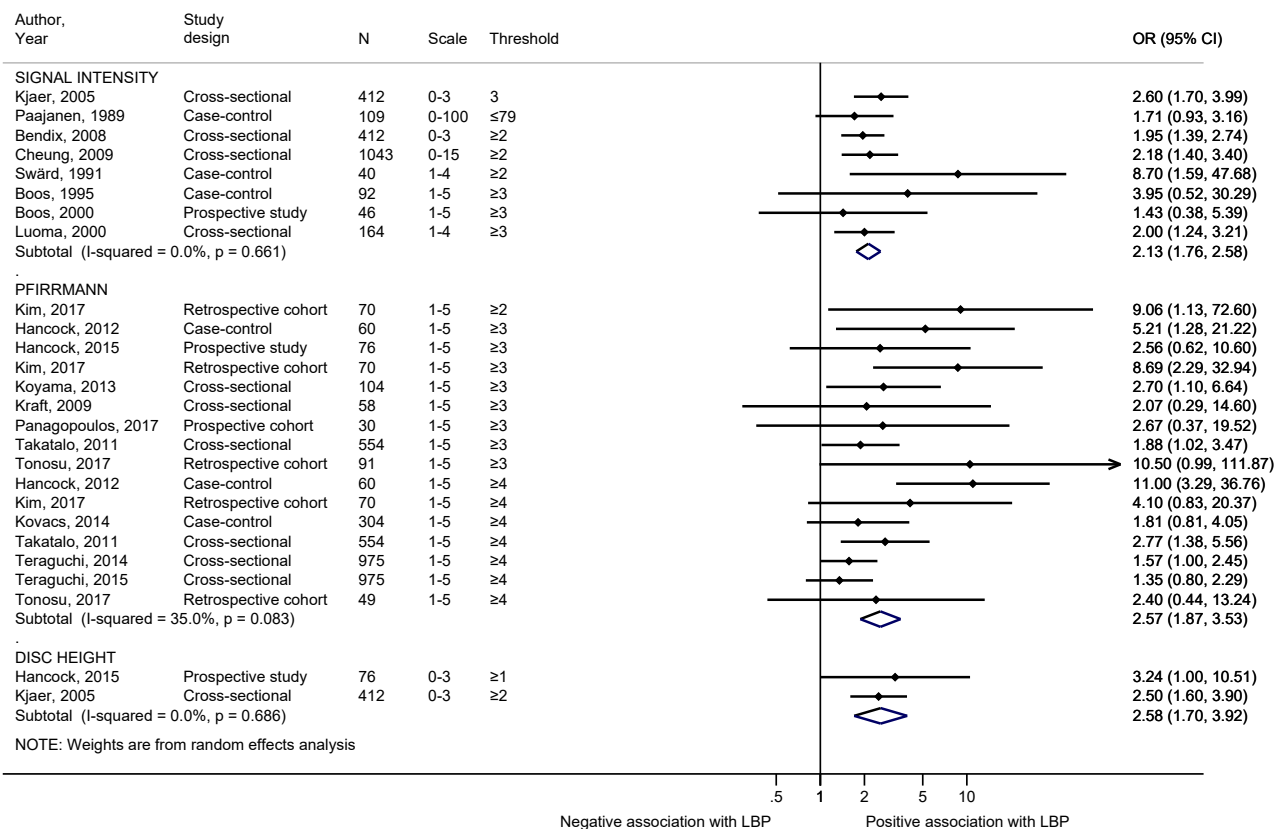

Supplement: Supplementary file 2 — Additional file 2. Forest plot. Associations between LBP and LDD (Disc Signal Intensity, Pfirrmann’s grade and Disc Height) in 20 studies. Associations are expressed as Odds Ratios with 95% confidence intervals (95% CI). [file 12891_2020_3268_MOESM2_ESM.pdf]
