# Supplementary material for: An exploratory study of different definitions and thresholds for lumbar disc degeneration assessed by MRI and their associations with low back pain using data from a cohort study of a general population
Source: BMC Musculoskelet Disord. 2020 Apr 17;21:253. doi: 10.1186/s12891-020-03268-4 (PMC7165403; doi:10.1186/s12891-020-03268-4)
Supplement: Supplementary file 3 — Additional file 3. Included studies. Study characteristics, definitions of LDD, thresholds for LDD and associations with LBP. Table of included studies in the review including study characteristics, definitions of LDD, thresholds for LDD and associations with LBP. (PDF 208 kb) [file 12891_2020_3268_MOESM3_ESM.pdf]

Additional file 3. Included studies. Study characteristics, definitions of LDD, thresholds for LDD and associations with LBP

Author and publication year	Study Design	Study Population and Population Characteristics	Definition of Disc Degeneration	Threshold MRI findings	Definition of LBP	Results Association btw. LDD and LBP OR (95% CI)
Bendix et al. 2008(24)	Cross-sectional MRI study of general population	General Population, Denmark.<br><br>412 40-year-old individuals from Danish cohort.<br><br>Females : 51.7%	**Signal Intensity**<br><br>0: Homogeneous Hyperintense (white)<br><br>1: Hyperintense with visible intranuclear cleft (white)<br><br>2: Intermediate signal intensity (grey)<br><br>3: Hypointense (black)<br>Eyre et al 1989(35)	0-1= No LDD<br><br>2-3 = LDD	LBP Month<br><br>-------------------------<br>LBP year	Black discs<br>1.20 (0.94;1.53)<br><br>Grey Discs<br>0.99 (0.85;1.15)<br><br>-------------------------<br>Black discs<br>1.95 (1.39;2.74)<br><br>Grey Discs<br>1.03 (0.87;1.20)
Boos et al. 1995(25)	Case-control study	92 Swiss Individuals<br><br>46 cases. LBP and sciatica severe enough for discectomy.<br>Age: Mean 35.2, range 20-50 years<br>Females: 26%<br><br>46 matched asymptomatic controls from general population<br>Age: Mean 36.2,	**Signal Intensity**<br><br>Grades I-V<br>Pearce et al. from Eyre 1989(35)	1-2 = No LDD<br><br>3-5 = LDD	LBP severe enough to require discectomy	3.95<br>(0.69;40.59) †

		range 20-50 years<br>Females: 26%				
Boos et al. 2000(26)	Prospective study on individuals with asymptomatic lumbar disc abnormalities detected in MRI	46 asymptomatic individuals from normal Swiss population(25) observed for an average of 5 years<br><br>Age: Mean 36.2, range 20-50 years<br>Females: 26%	**Signal Intensity**<br><br>Grades I-V<br>Pearce et al. from Eyre 1989(35)	1-2 = No LDD<br><br>3-5 = LDD	LBP at follow up: yes/no<br>-------------------------<br>LBP-Related Medical Consultation<br><br>-------------------------<br>LBP-Related Work Absence	1.43 (0.38;5.39)<br>-------------------------<br>2.21 (0.27;6.61)<br><br>-------------------------<br>4.63 (0.40;238.22)
Cheung et al. 2009(5)	Cross-sectional population study	1043 volunteers from general southern Chinese population.<br><br>Age: 18-55<br>Females: -	**Signal Intensity Schneiderman's classification**(7)<br><br>Signal Intensity Grades 0-3.<br>All 5 lumbar levels were scored (min. score= 0; max. score= 15)	0-1 = No LDD<br><br>2-15= LDD[1]	Pain in the low back for more than 2 weeks duration, sufficiently severe to require physician consultation or treatment	2.18 (1.40;3.40)
Hancock et al. 2012(12)	Case-control study	General Population in Australia.<br><br>30 cases<br>LBP with or without leg pain or sciatica<br>Age: Mean 36.8 (±7.4), range 18-50 years<br>Females: 47%<br><br>30 controls<br>Age: Mean 36.6 (±7.4), range 18-50	**Pfirrmann's grading system** (11)<br><br>Grades 1-5	1-2 = No LDD<br><br>3-5 = LDD<br><br>And<br><br>1-3 = No LDD<br><br>4-5 = LDD	At least moderate pain (measured using SF-36 question 7) of less than 6 weeks duration and no known or suspected serious spinal pathology. Patients needed to demonstrate centralisation of pain.	Assessor A<br><br>DD≥3:<br>5.21 (1.28;21.24)<br><br>DD≥4:<br>11.00 (3.29;36.75)<br>-------------------------<br>Assessor B<br><br>DD≥3:<br>5.09 (0.98;26.43)

		years<br>Females: 47%				DD≥4:<br>6.57 (2.11;20.48)
Hancock et al. 2015(13)	Prospective inception cohort study with 1-year follow up.	General Population in Australia.<br><br>76 people who had recovered from an episode of LBP within the previous 3 months.<br><br>Age 45.6 (±12.8) years<br><br>Females: 39.5%	**Pfirrmann's grading system**<br><br>Grades 1-5<br><br>--------------------------<br><br>**Disc Height**<br>Raininko et al. 1995(36)<br><br>0: Absent<br>1: Mild<br>2: Moderate<br>3: Severe	1-2 = no LDD<br><br>3-5 = LDD<br><br>--------------------------<br><br>≥ 1 = Disc height loss	Recurrence of LBP (at least 24 hours with a pain intensity of 3 or more on a 0-10 scale)<br><br>--------------------------<br>Recurrence of activity limiting LBP (moderate or greater activity limitation measured using an adaptation of Item 8 of the short form 36)<br>--------------------------<br>Recurrence of LBP	Multivariate analysis:<br>HR = 1.89 (0.42;8.53)<br><br>--------------------------<br>Multivariate analysis:<br>HR = 2.27 (0.28;18.26)<br><br>--------------------------<br>Univariate analysis:<br><br>≥3: HR = 2.56 (0.62;10.63)<br><br>Height loss:<br>≥ 1: HR=3.24 (1.0;10.52)
Kim et al. 2018(21)	Retrospective cohort study	70 young, Korean adults with L5 spondylolysis<br><br>Symptomatic group: 46 patients<br>Age: 32.1 (±5.2)<br>Females: 32.6%<br><br>Asymptomatic group: 24 patients<br>Age: 28.8 (±5.9)	**Pfirrmann's grading system**<br><br>Grades 1-5	1 = no LDD<br><br>2-5 = LDD<br>________________<br><br>1-2 = no LDD<br><br>3-5 = LDD<br>________________<br><br>1-3 = no LDD<br><br>4-5 = LDD	Low back pain or intermittent radiating pain	L4/L5:<br>≥2: 3.98 (1.10;14.69)†<br><br>≥3: 3.19 (0.92;12.65)†<br><br>L5/S1:<br>≥2: 9.06 (1.47;94.39)†

		Females: 20.8%		1-4 = no LDD<br><br>5 = LDD		≥3: 8.69 (2.42;34.78)†<br><br>≥4: 4.10 (0.98;24.19)†
Kjaer et al. 2005(27)	Cross-sectional cohort study of a general population	General population, in Denmark.<br><br>412 40-year-old individuals from Danish cohort<br><br>Females: 51.7%	**Disc Height (36)**<br><br>Grades 0-3<br><br>--------------------------<br>**Signal Intensity (35)**<br><br>Grades 0-3<br><br>--------------------------<br>**Nucleus shape (36)**<br><br>Grades 0-3	0-1 = no LDD<br><br>2-3 = LDD<br><br>--------------------------<br>0-2 = no LDD<br><br>3 = LDD<br><br>--------------------------<br>0-1 = no LDD<br><br>2-3 = LDD	LBP month<br>LBP year<br>Seeking Care<br>--------------------------<br>LBP month<br>LBP year<br>Seeking Care<br>--------------------------<br>LBP month<br>LBP year<br>Seeking Care	Disc Height:<br>1.8 (1.2;2.7)<br>2.5 (1.6;3.9)<br>2.0 (1.3;3.0)<br>--------------------------<br>Signal Intensity:<br>1.5 (1.0;2.2)<br>2.6 (1.7;4.0)<br>2.0 (1.3;3.2)<br>--------------------------<br>Nucleus shape:<br>1.8 (1.2;2.7)<br>2.5 (1.6;3.9)<br>2.0 (1.3;3.0)
Kovacs et al. 2014(14)	Case–control study	General population, in Spain.<br><br>240 Cases chronic LBP<br>Age: Median 43, Range 30-50 years<br>Females: 55%<br><br>64 Controls, asymptomatic<br>Age: Median 45, Range 30-50 years<br>Females: 45.3	**Pfirrmann's grading system**<br><br>Grades 1-5	1-3 = No LDD<br><br>4-5 = LDD	Chronic LBP (i.e., lasting ≥90 days), with or without leg pain	Crude analysis:<br><br>2.06 (1.05; 4.06)<br>-------------------------<br>Adjusted Analysis:<br><br>1.81 (0.81; 4.05)<br><br>(Adjusted by Modic changes and disc protrusion/herniation)

Koyama et al. 2013(15)	Cross-sectional study	104 Japanese collegiate gymnasts<br><br>Age: Mean 19.7 (±1) years<br><br>Females: 32.7%	**Pfirrmann's grading system**<br><br>Grades 1-5	1-2 = no LDD<br><br>3-5 = LDD	OCU test (pain during 10 specific activities. 0-2. Total score ≥1=LBP)	Crude analysis:<br>2.85 (1.27;6.42)<br>--------------------------<br>Adjusted analysis:<br>2.70 (1.10;6.66)<br><br>(Adjusted by limbus vertebra, weight, sex, and sporting experience)
Kraft et al. 2009(16)	Cross-sectional study	Elite riders and non-riding controls, Germany.<br>58 elite riders:<br>Age: Mean 32.4 (± 9.3) years<br>Females: 69%<br>30 non-riding controls:<br>Age: Mean 28.7 (±5.6) years<br>Females: 43.3%	**Pfirrmann's grading system**<br><br>Grades 1-5	1-2 = no LDD<br><br>3-5 = LDD	VAS 0-10	Riders:<br>2.07 (0.31;15.43) †<br><br>Controls:<br>1.91 (0.31;14.52) †
Luoma et al. 2000(28)	Cross-sectional MRI study	Working population, Finland (Cohort including Office workers Carpenters and Machine drivers)<br><br>164 Men<br>Age: 40-45 years	**Signal Intensity**<br><br>Grades 1-4<br><br>1: Bright<br>2: Grey<br>3: Dark<br>4: Black	1-2 = No LDD<br><br>3-4 = LDD	LBP 12-month prevalence<br><br>--------------------------<br>LBP 4-year prevalence	Crude analysis:<br>1.7 (1.1;2.5)<br><br>Adjusted analysis:<br>2.0 (1.2;3.1)<br>--------------------------<br>Crude analysis:<br>1.9 (1.2;3.1)<br><br>Adjusted analysis:<br>2.1 (1.2;3.6)

						Adjusted for height, history of car-driving, smoking, overweight
Paajanen et al. 1989 (29)	Case-control study	109 young, Finnish males.<br><br>75 cases, male conscripts referred to Central Military Hospital, Helsinki for LBP<br>Age: 20 (±1) years.<br><br>34 controls, healthy male volunteers.<br>Age: 20 (±0.6) years	**Signal Intensity**<br><br>Intensity was given a value of 100%. The degree of DD was calculated as the percentage decrease of signal intensity relative to this value.	80 - 100% = no LDD<br><br>0 – 79% = LDD[2]	Referred from military hospital for LBP	1.71 (0.95;3.24) †
Panagopoulos et al. 2017(22)	Exploratory prospective cohort study	General population<br><br>Cases: 20 individuals with recent-onset LBP.<br>Age: 37.4 (±9.4)<br>Females: 45%<br><br>Controls: 10 pain-free individuals.<br>Age: 39.8 (±9.4)<br>Female gender: 50%	**Pfirrmann's grading system**<br><br>Grades 1-5	1-2 = no LDD<br><br>3-5 = LDD	Recent-onset LBP of < 2 weeks' duration. Average pain intensity of ≥4 on an 11-point Numerical Pain Rating Scale 24 hours before the initial MRI; Sudden-onset episode of LBP (pain intensity increased by ≥4 points on the Numerical Pain Rating Scale during <24 hours)	2.67 (0.36;19.25)†
Swärd et al. 1991(30)	An MRI study Case-control study	24 Swedish male elite gymnasts with and without LBP<br>Age: 19-36, Median	**Signal intensity**<br><br>- No decrease<br>- Slight decrease	No decrease = No LDD	Previous or present LBP duration ≥1 week or recurrent	8.7 (1.66;49.87)†

		23 years<br><br>16 male non-athletes with and without LBP<br>Age: 23-36, Median 26 years	- Moderate decrease<br>- Severe decrease	Slight, moderate or severe decrease = LDD	pain irrespective of its duration.	
Takatalo et al. 2011(17)	A cross-sectional MRI study with questionnaires on low back pain and functional limitations	Northern Finland Birth Cohort (NFBC 1986)<br>554 individuals from the ʻNorthern Finland Birth Cohort 1986ʼ<br><br>Age: Mean 21.2, Range 20-23 years<br><br>Females: 42%	**Modified Pfirrmann's grading system**<br><br>Grades 1-5<br><br>Modified: Hyperintensity or isointensity of intervertebral disc to cerebrospinal fluid ratios were not used as criteria for Grades 1 through 3, because the cerebrospinal fluid was always hyperintense to the discs with the used MRI sequences.	1-2 = no LDD<br><br>3-5 = LDD<br><br>And<br><br>1-3 = no LDD<br><br>4-5= LDD	Six-month prevalence of LBP, NRS, frequency of LBP episodes, need for physician's consultations, need for pain medication and back-related functional limitations.<br><br>Clusters:<br>- No/minor symptoms<br>- Intermediate symptoms<br>- Major symptoms	DD ≥3:<br>Major Symptoms:<br>1.88 (1.02;3.48)<br><br>Intermediate Symptoms:<br><br>1.91 (0.96;3.79)<br>-------------------------<br>DD ≥ 4<br>Major Symptoms:<br>2.77 (1.38;5.57)<br><br>Intermediate Symptoms:<br>1.95 (0.84;4.53)
Teraguchi et al. 2014(18)	Cross-sectional study	General Population, Japan. Sub-cohort of the large-scale population-based cohort study Research on Osteoarthritis /Osteoporosis Against Disability (ROAD)<br><br>975 Individuals<br>Age: Mean 66.4(13.5), Range 21-	**Pfirrmann's grading system**<br><br>Grades 1-5	1-3 = No LDD<br><br>4-5 = LDD	LBP most days the past Month	Presence of DD<br>1.57 (1.02;2.49)<br>-------------------------<br><br>≥3 Degenerated discs:<br>1.75 (1.11;2.81)<br>-------------------------<br><br>1-2 Degenerated discs:<br>1.34 (0.84;2.20)

		97 years.<br>Females: 67%				
Teraguchi et al. 2015(19)	Cross-sectional population-based study in two regions of Japan.	General Population, in Japan. Sub-cohort of the large-scale population-based cohort study Research on Osteoarthritis /Osteoporosis Against Disability (ROAD)<br><br>975 Individuals<br>Age: Mean 66.4(13.5), Range 21-97 years.<br><br>Females: 67%	**Pfirrmann's grading system**<br><br>Grades 1-5	1-3 = No LDD<br><br>4-5 = LDD	LBP most days the past Month	Overall:<br>1.35 (0.8;2.3)<br>--------------------------<br>L1-L2:<br>1.0 (0.7;1.4)<br>--------------------------<br>L2-L3:<br>1.19 (0.9;1.6)<br>--------------------------<br>L3-L4:<br>1.17 (0.9;1.6)<br>--------------------------<br>L4-L5:<br>1.36 (0.9;1.9)<br>--------------------------<br>L5-S1:<br>1.14 (0.8;1.6)
Tonuso et al. 2017(20)	Retrospective cohort-study	Working Population, in Japan. Personnel at Kanto Rosai Hospital.<br><br>91 individuals without current LBP.<br>Age 34.9 (±10.6) years<br>Females: 52%	**Pfirrmann's grading system**<br><br>Grades 1-5	1-2 = No LDD<br><br>3-5 = LDD	Previous LBP - History of medical consultation for LBP	Univariate analysis:<br>12.7 (2.43;234.18)<br>--------------------------<br>Adjusted analysis:<br>10.5 (1.78;202.09)<br><br>(Adjusted for age and gender)
Tonosu et al. 2017(23)	Retrospective cohort-study	Working Population, in Japan. Personnel at Kanto Rosai Hospital. (10 year follow up)<br><br>49 individuals without current LBP.	**Pfirrmann's grading system**<br><br>Grades 1-5	1-3 = no LDD<br><br>4-5 = LDD	Previous LBP - History of medical consultation for LBP. pain localised between the costal margin and the inferior gluteal folds, as depicted in a diagram, with or	2.4 (0.42;12.78)

		Age 44.9 (±9.3) years<br>Females: 51%			without lower extremity pain in the past 1 month.	

† = OR and 95% CI calculated from prevalence of LDD findings in cases and controls. 1: 2-3 mild LDD; 4-15 Moderate to severe LDD. 2: 60-79% = intermediate loss of signal intensity. 0-59% = marked loss
MRI = Magnetic Resonance Imaging
LDD = Lumbar Disc Degeneration
LBP = Low Back Pain
