# Supplementary material for: An exploratory study of different definitions and thresholds for lumbar disc degeneration assessed by MRI and their associations with low back pain using data from a cohort study of a general population
Source: BMC Musculoskelet Disord. 2020 Apr 17;21:253. doi: 10.1186/s12891-020-03268-4 (PMC7165403; doi:10.1186/s12891-020-03268-4)
Supplement: Supplementary file 4 — Additional file 4. Accuracy of reduction in disc signal intensity at different thresholds as a predictor of LBP year at ages 41,45 and 49 years. Area under the curve values with 95% confidence intervals as measures of accuracy of reduction in disc signal intensity at different thresholds as a predictor of LBP year at ages 41,45 and 49 years. (PDF 366 kb) [file 12891_2020_3268_MOESM4_ESM.pdf]

Additional file 4.

Accuracy of reduction in disc signal intensity at different thresholds as a predictor of 'LBP year' at ages 41,45 and 49 years – Expressed as Areas Under the Curve (AUC) with 95% confidence intervals (95% CI). Note: The results for threshold ≥1 were underpowered and are therefore not presented in the Table.

**Disc signal intensity at 41 Years**	Threshold 2: ≥ grade 2 (n=356) (87.04%)<br><br>AUC (95% CI)	Threshold 3: = grade 3 (n=185) (45.23%)<br><br>AUC (95% CI)	P-value
**AUC (95% CI)**<br>**All Participants (n=409)**	**0.567 (0.505;0.629)**	**0.630 (0.572;0.689)**	**AUC T2= AUC T3: 0.020***
AUC (95% CI)<br>Men (n=197)	0.519 (0.456;0.583)	0.593 (0.535;0.651)	AUC T2= AUC T3: 0.017*
AUC (95% CI)<br>Women (n=210)	0.569 (0.508;0.631)	0.630 (0.571;0.688)	AUC T2= AUC T3: 0.016*
AUC (95% CI)<br>Upper lumbar spine (n=409)	0.542 (0.482;0.602)	0.565 (0.505;0.624)	AUC T2= AUC T3: 0.240
AUC (95% CI)<br>Lower lumbar spine (n=409)	0.583 (0.522;0.643)	0.625 (0.567;0.683)	AUC T2= AUC T3: 0.111
**Disc signal intensity at 45 Years**	Threshold 2: ≥ grade 2 (n=290) (85.04%)<br><br>AUC (95% CI)	Threshold 3: = grade 3 (n=170) (49.85%)<br><br>AUC (95% CI)	P-value
**AUC (95% CI)**<br>**All Participants (n=341)**	**0.586 (0.522;0.651)**	**0.554 (0.489;0.618)**	**AUC T2= AUC T3: 0.123**
AUC (95% CI)<br>Men (n=160)	0.586 (0.522;0.651)	0.550 (0.485;0.615)	AUC T2= AUC T3: 0.134

AUC (95% CI)<br>Women (n=181)	0.586 (0.521;0.650)	0.555 (0.490;0.619)	AUC T2= AUC T3: 0.267
AUC (95% CI)<br>Upper lumbar spine (n=341)	0.569 (0.506;0.633)	0.563 (0.500;0.628)	AUC T2= AUC T3: 0.767
AUC (95% CI)<br>Lower lumbar spine (n=341)	0.567 (0.502;0.632)	0.556 (0.491;0.620)	AUC T2= AUC T3: 0.589
**Disc signal intensity at 49 Years**	Threshold 2: ≥ grade 2 (n=251) (89.01%)<br>AUC (95% CI)	Threshold 3: = grade 3 (n=100) (35.46%)<br>AUC (95% CI)	P-value
**AUC (95% CI)<br>All Participants (n=282)**	**0.577 (0.502;0.652)**	**0.572 (0.502;0.642)**	**AUC T2= AUC T3: 0.859**
AUC (95% CI)<br>Men (n=132)	0.577 (0.502;0.652)	0.570 (0.499;0.641)	AUC T2= AUC T3: 0.825
AUC (95% CI)<br>Women (n=150)	0.582 (0.507;0.656)	0.541 (0.469;0.613)	AUC T2= AUC T3: 0.01*
AUC (95% CI)<br>Upper lumbar spine (n=282)	0.609 (0.538;0.679)	0.575 (0.505;0.644)	AUC T2= AUC T3: 0.247
AUC (95% CI)<br>Lower lumbar spine (n=282)	0.581 (0.507;0.656)	0.555 (0.484;0.627)	AUC T2= AUC T3: 0.324

*= P-value <0.05

--- = No participants with or without LDD at this threshold

LBP year = Low Back Pain during the last 12 months
