# Supplementary material for: An exploratory study of different definitions and thresholds for lumbar disc degeneration assessed by MRI and their associations with low back pain using data from a cohort study of a general population
Source: BMC Musculoskelet Disord. 2020 Apr 17;21:253. doi: 10.1186/s12891-020-03268-4 (PMC7165403; doi:10.1186/s12891-020-03268-4)
Supplement: Supplementary file 5 — Additional file 5. Accuracy of reduction in disc height at different thresholds as a predictor of LBP year at ages 41,45 and 49 years. Area under the curve values with 95% confidence intervals as measures of accuracy of reduction in disc height at different thresholds as a predictor of LBP year at ages 41, 45 and 49 years. [file 12891_2020_3268_MOESM5_ESM.pdf]

Additional file 5.

Accuracy of reduction in disc height at different thresholds as a predictor of 'LBP year' at ages 41,45 and 49 years – Expressed as Areas Under the Curve (AUC) with 95% confidence intervals (95% CI).

**Disc height at 41 Years**	Threshold 1: ≥ grade 1(n=319) (77.99%)<br>AUC (95% CI)	Threshold 2: ≥ grade 2 (n=218) (53.30%)<br>AUC (95% CI)	Threshold 3: = grade 3 (n=12) (2.93%)<br>AUC (95% CI)	P-value
**AUC (95% CI)**<br>**All Participants (n=409)**	**0.557 (0.496;0.618)**	**0.627 (0.568;0.687)**	**0.551 (0.491;0.612)**	**AUC T1=AUC T2: 0.004***<br>**AUC T2= AUC T3: 0.006***<br>**AUC T1= AUC T3: 0.760**
AUC (95% CI)<br>Men (n=197)	0.514 (0.452;0.575)	0.602 (0.544;0.661)	0.467 (0.407;0.528)	AUC T1=AUC T2: 0.001*<br>AUC T2= AUC T3: 0.000*<br>AUC T1= AUC T3: 0.121
AUC (95% CI)<br>Women (n=210)	0.546 (0.485;0.606)	0.612 (0.551;0.672)	0.550 (0.489;0.610)	AUC T1=AUC T2: 0.001*<br>AUC T2= AUC T3: 0.004*<br>AUC T1= AUC T3: 0.586
AUC (95% CI)<br>Upper lumbar spine (n=409)	0.562 (0.503;0.621)	0.595 (0.537;0.652)	---	AUC T1=AUC T2: 0.097
AUC (95% CI)<br>Lower lumbar spine (n=409)	0.562 (0.501;0.623)	0.618 (0.559;0.678)	0.548 (0.487;0.608)	AUC T1=AUC T2: 0.019<br>AUC T2= AUC T3: 0.01*<br>AUC T1= AUC T3: 0.519
**Disc height at 45 Years**	Threshold 1: ≥ grade 1 (n=237) (69.50%)<br>AUC (95% CI)	Threshold 2: ≥ grade 2 (n=168) (49.27%)<br>AUC (95% CI)	Threshold 3: = grade 3 (n=14) (4.11%)<br>AUC (95% CI)	P value
**AUC (95% CI)**<br>**All Participants (n=341)**	**0.554 (0.490;0.618)**	**0.556 (0.492;0.620)**	**0.572 (0.507;0.636)**	**AUC T1=AUC T2: 0.724**<br>**AUC T2= AUC T3: 0.296**<br>**AUC T1= AUC T3: 0.275**

AUC (95% CI)<br>Men (n=160)	0.546 (0.482;0.610)	0.553 (0.488;0.617)	0.552 (0.488;0.616)	AUC T1=AUC T2: 0.725<br>AUC T2= AUC T3: 0.954<br>AUC T1= AUC T3: 0.795
AUC (95% CI)<br>Women (n=181)	0.559 (0.494;0.623)	0.558 (0.493;0.622)	0.572 (0.508;0.637)	AUC T1=AUC T2: 0.929<br>AUC T2= AUC T3: 0.441<br>AUC T1= AUC T3: 0.540
AUC (95% CI)<br>Upper lumbar spine (n=341)	0.574 (0.510;0.638)	0.580 (0.516;0.643)	---	AUC T1=AUC T2: 0.735
AUC (95% CI)<br>Lower lumbar spine (n=341)	0.556 (0.492;0.620)	0.556 (0.491;0.620)	0.572 (0.507;0.636)	AUC T1=AUC T2: 0.896<br>AUC T2= AUC T3: 0.284<br>AUC T1= AUC T3: 0.302
**Disc height at 49 Years**	Threshold 1: ≥ grade 1 (n=238) (84.40%)<br>AUC (95% CI)	Threshold 2: ≥ grade 2 (n=187) (66.31%)<br>AUC (95% CI)	Threshold 3: = grade 3 (n=29) (10.28%)<br>AUC (95% CI)	P value
**AUC (95% CI)**<br>**All Participants (n=282)**	**0.596 (0.523;0.670)**	**0.596 (0.523;0.669)**	**0.544 (0.471;0.616)**	**AUC T1=AUC T2: 0.995**<br>**AUC T2= AUC T3: 0.134**<br>**AUC T1= AUC T3: 0.070**
AUC (95% CI)<br>Men (n=132)	0.596 (0.523;0.670)	0.592 (0.519;0.665)	0.527 (0.455;0.599)	AUC T1=AUC T2: 0.864<br>AUC T2= AUC T3: 0.046*<br>AUC T1= AUC T3: 0.019*
AUC (95% CI)<br>Women (n=150)	0.597 (0.524;0.670)	0.600 (0.528;0.672)	0.536 (0.464;0.608)	AUC T1=AUC T2: 0.876<br>AUC T2= AUC T3: 0.081<br>AUC T1= AUC T3: 0.074
AUC (95% CI)<br>Upper lumbar spine (n=282)	0.658 (0.592;0.724)	0.643 (0.577;0.709)	---	AUC T1=AUC T2: 0.448

AUC (95% CI)<br>Lower lumbar spine (n=282)	0.582 (0.509;0.655)	0.583 (0.510;0.655)	0.544 (0.471;0.616)	AUC T1=AUC T2: 0.971<br>AUC T2= AUC T3: 0.233<br>AUC T1= AUC T3: 0.202
*= P-value <0.05<br>--- = No participants with or without LDD at this threshold<br>LBP = Low back Pain during the last 12 months				
