# Supplementary material for: An exploratory study of different definitions and thresholds for lumbar disc degeneration assessed by MRI and their associations with low back pain using data from a cohort study of a general population
Source: BMC Musculoskelet Disord. 2020 Apr 17;21:253. doi: 10.1186/s12891-020-03268-4 (PMC7165403; doi:10.1186/s12891-020-03268-4)
Supplement: Supplementary file 6 — Additional file 6. STROBE statement- checklist of items that should be included in reports of observational studies. Completed STROBE checklist. [file 12891_2020_3268_MOESM6_ESM.pdf]

STROBE Statement—checklist of items that should be included in reports of observational studies

	Item No	Recommendation
**Title and abstract**	1	(*a*) Indicate the study's design with a commonly used term in the title or the abstract<br>**-Title, page 1**<br>**-Abstract, page 3**
		(*b*) Provide in the abstract an informative and balanced summary of what was done and what was found<br>**-Abstract, page 3**
**Introduction**		
Background/rationale	2	Explain the scientific background and rationale for the investigation being reported<br>**-Background, page 5-6**
Objectives	3	State specific objectives, including any prespecified hypotheses<br>**-Background, page 6 (aim)**
**Methods**		
Study design	4	Present key elements of study design early in the paper<br>**-Methods, Study design, page 7**
Setting	5	Describe the setting, locations, and relevant dates, including periods of recruitment, exposure, follow-up, and data collection<br>**-Methods, Study participants and sampling from "Backs on Funen", page 7**
Participants	6	(*a*) *Cohort study*—Give the eligibility criteria, and the sources and methods of selection of participants. Describe methods of follow-up<br>*Case-control study*—Give the eligibility criteria, and the sources and methods of case ascertainment and control selection. Give the rationale for the choice of cases and controls<br>*Cross-sectional study*—Give the eligibility criteria, and the sources and methods of selection of participants<br>**-Methods, Study procedures of "Backs on Funen", page 7**
		(*b*) *Cohort study*—For matched studies, give matching criteria and number of exposed and unexposed<br>*Case-control study*—For matched studies, give matching criteria and the number of controls per case<br>**N/A**
Variables	7	Clearly define all outcomes, exposures, predictors, potential confounders, and effect modifiers. Give diagnostic criteria, if applicable<br>**-Methods, LBP variables, page 8**<br>**-Methods, Definitions of MRI variables, page 8**<br>**-Methods, Other variables, page 9**
Data sources/ measurement	8*	For each variable of interest, give sources of data and details of methods of assessment (measurement). Describe comparability of assessment methods if there is more than one group<br>**-Methods, LBP variables, page 8**<br>**-Methods, Definitions of MRI variables, page 8**
Bias	9	Describe any efforts to address potential sources of bias<br>**-Methods, Study procedures of "Backs on Funen", page 7**
Study size	10	Explain how the study size was arrived at

		**-Methods, Study participants and sampling from "Backs on Funen", page 7**
Quantitative variables	11	Explain how quantitative variables were handled in the analyses. If applicable, describe which groupings were chosen and why<br>**-Methods, LBP variables, page 8**<br>**-Methods, Definitions of MRI variables, page 8**
Statistical methods	12	(*a*) Describe all statistical methods, including those used to control for confounding<br>-**Methods, statistical analyses, page 9-10**
		(*b*) Describe any methods used to examine subgroups and interactions<br>**Methods, statistical analyses, page 10 (secondary analyses)**
		(*c*) Explain how missing data were addressed<br>**Methods, Missing data, page 10**
		(*d*) *Cohort study*—If applicable, explain how loss to follow-up was addressed<br>*Case-control study*—If applicable, explain how matching of cases and controls was addressed<br>*Cross-sectional study*—If applicable, describe analytical methods taking account of sampling strategy<br>**-N/A**
		(*e*) Describe any sensitivity analyses<br>**-N/A**

Continued on next page

Results		
Participants	13*	(a) Report numbers of individuals at each stage of study—eg numbers potentially eligible, examined for eligibility, confirmed eligible, included in the study, completing follow-up, and analysed<br>-**Results, Study participants, page 10**<br>**-Table 1**
		(b) Give reasons for non-participation at each stage<br>**-N/A**
		(c) Consider use of a flow diagram<br>**-N/A**
Descriptive data	14*	(a) Give characteristics of study participants (eg demographic, clinical, social) and information on exposures and potential confounders<br>-**Results, Study participants, page 10**<br>-**Results, Prevalence of MRI findings, page 11**<br>**-Table 1**
		(b) Indicate number of participants with missing data for each variable of interest<br>**-Results, Missing data, page 11**
		(c) *Cohort study*—Summarise follow-up time (eg, average and total amount)<br>**-Results, Study participants, page 10**
Outcome data	15*	*Cohort study*—Report numbers of outcome events or summary measures over time
		Case-control study—Report numbers in each exposure category, or summary measures of exposure
		Cross-sectional study—Report numbers of outcome events or summary measures<br>**-Results, Study participants, page 10**<br>**-Table 1**
Main results	16	(*a*) Give unadjusted estimates and, if applicable, confounder-adjusted estimates and their precision (eg, 95% confidence interval). Make clear which confounders were adjusted for and why they were included<br>**-Table 2 and 3**<br>**-Results, Associations between Disc signal intensity and LBP, page 15**<br>**-Results, Associations between Disc height and LBP, page 15-16**
		(*b*) Report category boundaries when continuous variables were categorized<br>-**N/A**
		(*c*) If relevant, consider translating estimates of relative risk into absolute risk for a meaningful time period<br>-**N/A**
Other analyses	17	Report other analyses done—eg analyses of subgroups and interactions, and sensitivity analyses<br>**-Table 2 and 3**<br>**-Results, Associations between Disc signal intensity and LBP, page 15**<br>**-Results, Associations between Disc height and LBP, page 15-16**
**Discussion**		
Key results	18	Summarise key results with reference to study objectives<br>**-Discussion, Summary of main findings, page 16**
Limitations	19	Discuss limitations of the study, taking into account sources of potential bias or imprecision. Discuss both direction and magnitude of any potential bias

		**-Discussion, Limitations, page 18-19**
Interpretation	20	Give a cautious overall interpretation of results considering objectives, limitations, multiplicity of analyses, results from similar studies, and other relevant evidence<br>**-Discussion, Interpretation, page 17-18**
Generalisability	21	Discuss the generalisability (external validity) of the study results<br>**-Discussion, Interpretation, page 17-18**
**Other information**		
Funding	22	Give the source of funding and the role of the funders for the present study and, if applicable, for the original study on which the present article is based<br>**-Declarations, Funding, page 21**

*Give information separately for cases and controls in case-control studies and, if applicable, for exposed and unexposed groups in cohort and cross-sectional studies.

Note: An Explanation and Elaboration article discusses each checklist item and gives methodological background and published examples of transparent reporting. The STROBE checklist is best used in conjunction with this article (freely available on the Web sites of PLoS Medicine at http://www.plosmedicine.org/, Annals of Internal Medicine at http://www.annals.org/, and Epidemiology at http://www.epidem.com/). Information on the STROBE Initiative is available at www.strobe-statement.org.
